# Internalization and Transportation of Endothelial Cell Surface K_Ca_2.3 and K_Ca_3.1 in Normal Pregnancy and Preeclampsia

**DOI:** 10.1155/2019/5820839

**Published:** 2019-11-23

**Authors:** Shinkyu Choi, Ji Aee Kim, Seikwan Oh, Mi Hye Park, Geum Joon Cho, Suk Hyo Suh

**Affiliations:** ^1^Department of Physiology, Medical School, Ewha Womans University, Seoul 07985, Republic of Korea; ^2^Department of Molecular Medicine, Medical School, Ewha Womans University, Seoul 07985, Republic of Korea; ^3^Department of Obstetrics and Gynecology, Medical School, Ewha Womans University, Seoul 07985, Republic of Korea; ^4^Department of Obstetrics and Gynecology, Medical School, Korea University, Seoul 08308, Republic of Korea

## Abstract

Altered redox state modulates the expression levels of endothelial K_Ca_2.3 and K_Ca_3.1 (K_Ca_s) in normal pregnancy (NP) and preeclampsia (PE), thereby regulating vascular contractility. The mechanisms underlying K_Ca_s endocytosis and transportation remain unknown. We investigated the regulation of K_Ca_s expression in plasma membrane (PM) during NP and PE. Cultured human uterine artery endothelial cells were incubated in serum from normal nonpregnant women and women with NP or PE, or in oxidized LDL-, or lysophosphatidylcholine- (LPC-) containing a medium for 24 hours. NP serum elevated PM levels of K_Ca_s and reduced caveolin-1 and clathrin levels. PE serum, oxidized LDL, or LPC reduced PM levels of K_Ca_s and elevated caveolin-1, clathrin, Rab5c, and early endosome antigen-1 (EEA1) levels. Reduced K_Ca_s levels by PE serum or LPC were reversed by inhibition of caveolin-1, clathrin, or EEA1. Catalase and glutathione peroxidase 1 (GPX1) knockdown elevated PM-localized K_Ca_s levels and reduced caveolin-1 and clathrin levels. Elevated K_Ca_2.3 levels upon catalase and GPX1 knockdown were reversed by PEG-catalase treatment. An H_2_O_2_ donor reduced clathrin and Rab5c. In contrast, elevated clathrin, caveolin-1, or colocalization of caveolin-1 with K_Ca_3.1 by PE serum or LPC was reversed by NADPH oxidase inhibitors or antioxidants. A superoxide donor xanthine+xanthine oxidase elevated caveolin-1 or Rab5c levels. We concluded that K_Ca_s are endocytosed in a caveola- or a clathrin-dependent manner and transported in a Rab5c- and EEA1-dependent manner during pregnancy. The endocytosis and transportation processes may slow down via H_2_O_2_-mediated pathways in NP and may be accelerated via superoxide-mediated pathways in PE.

## 1. Introduction

K_Ca_2.3 and K_Ca_3.1 play an important role in endothelial control of vascular contractility. Activation of these K^+^ channels induces K^+^ efflux and endothelial hyperpolarization, which hyperpolarize vascular smooth muscle cells (VSMCs) by activating inward-rectifier K^+^ channels and spreading to VSMCs through gap junctions, respectively [1–3]. In addition, endothelial hyperpolarization enhances Ca^2+^ entry through Ca^2+^-permeable channels such as transient receptor potential channels by increasing its electrical driving force and elevates intracellular Ca^2+^ levels [4], which stimulates nitric oxide (NO) production in endothelial cells (ECs) [5]. NO and VSM hyperpolarization relax blood vessels, thereby controlling vascular contractility. The contribution of NO and VSM hyperpolarization to the control of vascular contractility might vary between conduit arteries and resistant arteries. The contribution of NO was most prominent in the aorta, whereas that of VSM hyperpolarization was most prominent in the distal mesenteric arteries, suggesting that VSM hyperpolarization plays a more important role in the control of vasorelaxation in resistant arteries than in conduit arteries [6–9]. Since resistant arteries are the main regulators of systemic vascular resistance, endothelial K_Ca_2.3 and K_Ca_3.1 might play an important role in the regulation of blood pressure.

Since endothelial hyperpolarization is caused by K_Ca_2.3 and K_Ca_3.1 activation, the magnitude of endothelial hyperpolarization may be affected by the expression levels of these K^+^ channels in plasma membrane (PM). Thus, K_Ca_2.3 and K_Ca_3.1 upregulation might increase endothelial hyperpolarization, thereby potentiating L-NAME-resistant, K_Ca_2.3 and K_Ca_3.1 activation-induced endothelium-dependent relaxation, as shown in blood vessels during aging process and normal pregnancy (NP) [10, 11]. On the contrary, downregulation of K_Ca_2.3 and K_Ca_3.1 activity and expression causes endothelial dysfunction, thereby developing vascular diseases, such as preeclampsia (PE) and Fabry disease [10, 12–14].

K_Ca_2.3 and K_Ca_3.1 levels are affected by various stimuli, such as sphingolipids and redox state. K_Ca_3.1 upregulation was caused by an altered sphingolipid profile via a H_2_O_2_/Fyn-mediated pathway during the aging process, whereas globotriaosylceramide downregulated K_Ca_3.1 in Fabry disease [14]. K_Ca_2.3 and K_Ca_3.1 levels were regulated by the altered redox state in pregnancy [10]. K_Ca_2.3 and K_Ca_3.1 were upregulated by soluble serum factors, such as vascular endothelial growth factor (VEGF) in NP. H_2_O_2_ plays an important role in K_Ca_2.3 and K_Ca_3.1 upregulation during aging [11] and NP [10]. On the other hand, K_Ca_2.3 and K_Ca_3.1 were downregulated by serum factors, such as progesterone and oxidized low-density lipoprotein (LDL) through superoxide generation in PE [10].

Our previous study explains how membrane levels of K_Ca_3.1 are reduced in Fabry disease. Clathrin-dependent internalization, Rab5c, early endosome antigen-1- (EEA1-) dependent transportation, and lysosomal degradation were involved in globotriaosylceramide-induced K_Ca_3.1 downregulation in Fabry disease [12]. However, little is known about how membrane levels of K_Ca_2.3 and K_Ca_3.1 are regulated in NP and PE. Thus, we investigated the regulation of K_Ca_2.3 and K_Ca_3.1 expression in PM during NP and PE and found that endothelial K_Ca_2.3 and K_Ca_3.1 are internalized from PM via caveola- or clathrin-dependent pathways. Rab5c and early EEA1 were involved in transportation of the internalized K^+^ channel proteins. Moreover, K_Ca_2.3 and K_Ca_3.1 internalization and transportation processes were delayed in NP and facilitated in PE.

## 2. Materials and Methods

Studies involving human subjects were approved by the local ethics committee, the Institutional Review Board of the Ewha Womans University Mokdong Hospital, and Korea University Guro Hospital and were conducted in accordance with the Declaration of Helsinki. All patients provided their written informed consent prior to the inclusion in this study. Experiments with mice were approved by the local ethics committee and the Institutional Review Board of the Ewha Womans University Mokdong Hospital and were conducted in accordance with the Declaration of Helsinki, the Animal Care Guidelines of the Ewha Womans University, Medical School, and the National Institutes of Health Guide for the Care and Use of Laboratory Animals.

### 2.1. Human Subjects

The study population consisted of Asian women who were not pregnant or had either a NP or PE (Table 1). Pregnancies were considered normal when patients did not have medical and obstetric complications of pregnancy and delivered a newborn at a gestational age of 37-42 weeks. Preeclampsia was defined by a systolic blood pressure over 140 mmHg and a diastolic blood pressure over 90 mmHg after 20 weeks of gestation in a previously normotensive woman, and the onset of proteinuria exceeding 300 mg of protein during 24 hours of urine collection. Nonpregnant women were healthy premenopausal volunteers taking no medications. Preeclamptic patients and normal pregnant women were matched for age (±3 years) and gestational age (±2 weeks), and nonpregnant healthy female volunteers were matched for age (±3 years). Blood samples were obtained from subjects during the third trimester of pregnancy. The study population was monitored at the Department of Obstetrics and Gynecology from the first trimester until their pregnancy was completed without complications. Exclusion criteria included the following: altered renal function, diabetes or chronic diseases, twin pregnancies, recurrent miscarriages, fetal growth retardation, and *abruptio* placenta. Smokers and women with a history of essential hypertension were also excluded from this study. Gestational age was defined as the interval between the first day of the mother's last menstrual period and the date of delivery.

### 2.2. Animals and Tissue Collection

We studied young C57BL/6 wild-type mice (about 20-week-old; *n* = 24), and catalase/glutathione peroxidase 1 (GPX1) double knockout (catalase^−/−^/GPX1^−/−^) mice (about 20-week-old; n = 18), generously donated by Dr. Ye-Shih Ho (Wayne State Medical School, Detroit, MI) [15]. Mice were anesthetized by an intraperitoneal injection of pentobarbital sodium (50 mg/kg body weight) and sacrificed by cervical dislocation.

### 2.3. Cell Culture and Serum Treatment

Human uterine microvascular ECs (HUtMECs), which were purchased from PromoCell GmbH (Heidelberg, Germany), were maintained in EC Growth Medium MV2 (PromoCell GmbH). For serum treatment, HUtMECs were plated in 6-well plates for 24 hours. The concentration of fetal bovine serum in a culture medium was gradually decreased from 10% to 5, 2, and 0% over 30 minutes, and HUtMECs were incubated in a serum-free medium for 30 minutes. After that, a culture medium was substituted with serum from normal nonpregnancy (NNP) women or women with NP or PE, and the cells were incubated for 24 hours.

Mouse aortic endothelial cells (MAECs) were isolated from the mouse aortas as described [16]. Briefly, periadventitial fats and connective tissues around the aorta were carefully cleaned in Ca^2+^-free phosphate-buffered saline under a dissecting microscope. Matrigel (BD Biosciences, San Jose, CA) was plated and polymerized at 37°C for 30 minutes. After that, aorta pieces were placed with the intima side down on the Matrigel. To demonstrate the endothelial nature of the cell, 1,1′-dioctadecyl-3,3,3′,3′-tetramethyl-indocarbocyanine perchlorate-labeled acetylated low-density lipoprotein (Biomedical Technologies Inc., Stoughton, MA) uptake assay was employed. MAECs were used within 2 passages and not above 3 passages.

### 2.4. Immunoblotting and Immunoprecipitation

For immunoblotting, cell lysates were used to examine the protein level. After proper processing of each type of sample, total protein was measured using the bicinchoninic acid assay (Pierce Biotechnology, Rockford, IL). The same amount of total protein was analyzed using SDS-PAGE on 7.5–12% gels and transferred to nitrocellulose membrane (Invitrogen, Eugene, OR). Membranes were blocked for 1 hour in 5% bovine serum albumin in Tris-buffered saline with 0.1% Tween-20 and incubated overnight at 4°C with primary antibodies (Abs) diluted in blocking buffer. Membranes were then washed three times with Tris-buffered saline with 0.1% Tween-20 and incubated for 1 hour with horseradish peroxidase-conjugated secondary Abs diluted in blocking buffer. The immunoblots were visualized by chemiluminescence reagents bought from GE Healthcare (Piscataway, NJ). Data processing was performed using a luminescent image analyzer LAS-3000 (Fujifilm, Tokyo, Japan) and IMAGE GAUSE software.

For immunoprecipitation, cells were washed twice with phosphate buffer saline and lysed in lysis buffer containing protease inhibitor cocktail (Sigma-Aldrich, St. Louis, MO) at 4°C for 1 hour. The lysate samples were prepared by centrifugation at 12000 × g for 30 minutes to eliminate the cell debris. Total protein concentration was estimated as described above. The lysate samples were precleared with a nonspecific IgG Ab. Total 30 *μ*L of 50% protein G-coupled dynabead slurry (Invitrogen) was added to an Ab (1-5 *μ*g) diluted in 200 *μ*L phosphate buffer saline with Tween 20, incubated for 15 minutes at room temperature (about 20°C) with rotating, and washed. Precleared lysate samples were incubated with the dynabeads-Ab by rotating at 4°C overnight or at room temperature for 30 minutes. Following that, the dynabeads-antigen-Ab complexes were washed three times with the lysis buffer, and the antigen was eluted in 2x SDS-PAGE sample buffer by heating at 70°C for 10 minutes. Immunoprecipitates were separated using 7.5–12% SDS-PAGE and analyzed by immunoblotting.

### 2.5. Biotinylation of Cell Surface Protein

Cells were washed twice with phosphate buffer saline and labeled with 1 mM sulfosuccinimidyl-2-(biotinamido)ethyl-1, 3-dithiopropionate (EZ-Link-sulfo-NHS-SS-biotin; Pierce Biotechnology) in labeling buffer (150 mM NaCl, 20 mM HEPES, 3 mM CaCl_2_, and 1 mM MgCl_2_) for 30 minutes to 1 hour at room temperature. After the cells were washed, any nonreacted biotinylation reagent was quenched with 100 mM glycine, the cells were lysed in NP40 lysis buffer, and the proteins were incubated with 30 *μ*L of dynabead M-280 streptavidin (Invitrogen) for 3 hours at 4°C with rotation and then washed three times with lysis buffer. The proteins were eluted from streptavidin bead in 2x SDS-PAGE sample buffer by heating at 65°C for 5 minutes. Supernatant was subjected to 7.5–12% SDS-PAGE and analyzed by immunoblotting.

### 2.6. siRNA Transfection

Negative control siRNAs (SN-1012) and siRNAs against EEA1 (SDH-1001) were purchased from Bioneer (Daejeon, Korea). Negative control siRNAs (sc-36869) and siRNAs against NADPH oxidase 4 (NOX4; sc-41586) were purchased from Santa Cruz Biotechnology. ECs were transiently transfected with the siRNAs using an siRNA transfection reagent (Santa Cruz Biotechnology) according to the procedure suggested by the manufacturer. Cell lysates were prepared 24 hours after transfection, and immunoblotting was performed using anti-EEA1 Abs.

### 2.7. Electrophysiology

The patch-clamp technique was used in whole-cell configurations at 20-22°C. Whole-cell currents were measured using ruptured patches and monitored in voltage-clamp modes with an EPC-9 (HEKA Elektronik, Lambrecht, Germany). The holding potential was 0 mV, and currents were monitored by the repetitive application of voltage ramps from −100 to +100 mV with a 10-second interval (sampling interval 0.5 milliseconds, 650 millisecond duration). The standard external solution contained (in mM) 150 NaCl, 6 KCl, 1.5 CaCl_2_, 1 MgCl_2_, 10 HEPES, and 10 glucose, pH adjusted to 7.4 with NaOH. The pipette solution for whole-cell recording contained (in mM) 40 KCl, 100 K-aspartate, 2 MgCl_2_, 0.1 EGTA, 4 Na_2_ATP, and 10 HEPES, pH adjusted to 7.2 with KOH. To buffer free Ca^2+^, the appropriate amount of Ca^2+^ (calculated using CaBuf software; G. Droogmans, Leuven, Belgium) was added in the presence of 5 mM EGTA.

K_Ca_3.1 currents were activated by loading 1 *μ*M Ca^2+^ via a patch pipette in whole-cell clamped MAECs. K_Ca_3.1 current was normalized to cell capacitance, and the selective K_Ca_3.1 blocker TRAM-34-sensitive current was measured as the K_Ca_3.1 current.

### 2.8. Reagents

Reagents were purchased from Sigma-Aldrich (St. Louis, MO) and dissolved in sterilized distilled water unless indicated otherwise. The cells were treated with xanthine+xanthine oxidase (X/XO), tert-butylhydroperoxide (TBHP), or oxidized LDL (Intracel Inc., Frederick, MD) or the major component of oxidized LDL, lysophosphatidylcholine (LPC; dissolved in chloroform : methanol, 2 : 1) for 24 hours. Abs specific to lectin-like oxidized LDL receptor 1 (LOX1; ab81709, Abcam, Cambridge, MA), chlorpromazine (CPZ; C8138), or methyl-*β*-cyclodextrin (M*β*CD; C4555) were pretreated for 1 hour. Abs against K_Ca_2.3 (sc-28621), K_Ca_3.1 (sc-32949), GAPDH (sc-25778), and *β*-actin (sc-130656) were purchased from Santa Cruz Biotechnology (Santa Cruz, CA).

The final concentration of DMSO, chloroform, or methanol in media was less than 0.1%, and these solvents did not have any effect on the experiments tested in this study (data not shown).

### 2.9. Statistics

Data represent mean ± SEM. To prove the statistical significance between groups, one-way ANOVA with Bonferroni's post hoc or 2-tailed Student's *t*-test was used. A *P* value of 0.05 or lower was considered statistically significant. Calculations were performed with SPSS 14.0 for Windows (SPSS, Chicago, IL).

## 3. Results

### 3.1. Endothelial Membrane Levels of K_Ca_2.3 and K_Ca_3.1 Are Altered during Pregnancy

**Figure 1:**
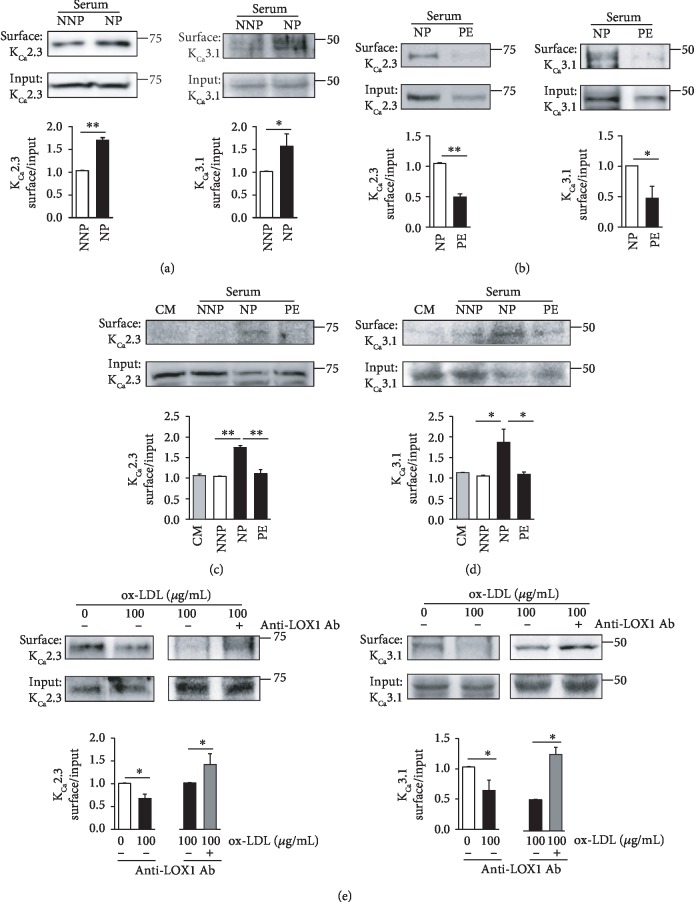
Membrane levels of K_Ca_2.3 and K_Ca_3.1 are altered in NP and PE. (a) PM levels of K_Ca_2.3 and K_Ca_3.1 in ECs treated with NNP or NP serum. (b) PM levels of K_Ca_2.3 and K_Ca_3.1 in ECs treated with NP or PE serum. (c, d) PM levels of K_Ca_2.3 (c) and K_Ca_3.1 (d) in ECs treated with CM, NNP serum, NP serum, or PE serum. (e) PM levels of K_Ca_2.3 or K_Ca_3.1 in ECs treated with oxidized LDL. Reduced K_Ca_2.3 or K_Ca_3.1 levels were reversed by blocking oxidized LDL receptor using an anti-LOX1 Ab. Blots shown are representative of the three to five experiments performed with three to five different cultures. Bar graphs represent the mean ± SEM. ^∗^*P* < 0.05, ^∗∗^*P* < 0.01.

**Figure 2:**
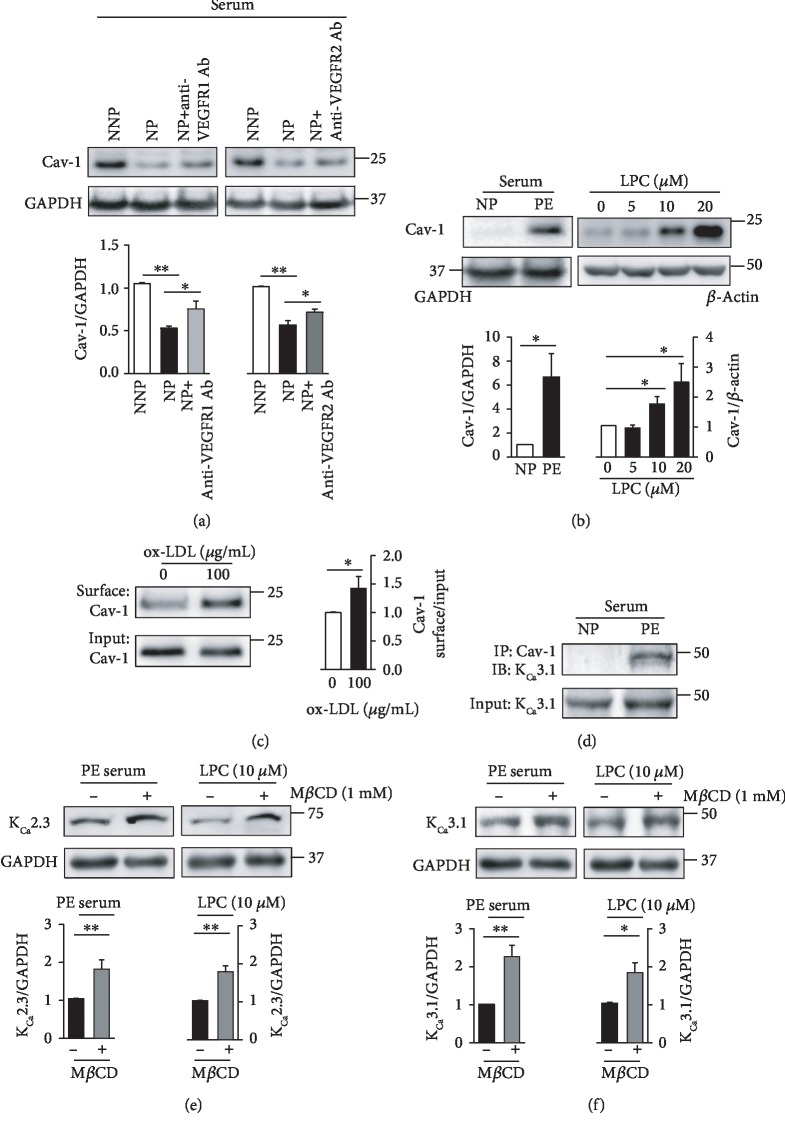
Cav-1 is necessary for K_Ca_2.3 and K_Ca_3.1 internalization from the PM. (a) Cav-1 levels were reduced in ECs treated with NP serum, which were reversed by VEGFR inhibition using anti-VEGFR1 and anti-VEGFR2 Abs. (b) Cav-1 levels in ECs treated with NP serum or PE serum, and in ECs treated with LPC. LPC enhanced Cav-1 levels in ECs in a concentration-dependent manner. (c) PM localization of Cav-1 was increased by oxidized LDL. (d) Coimmunoprecipitation showing the interaction between Cav-1 and K_Ca_3.1 in ECs treated with NP or PE serum. Input is lysate without primary Ab. (e, f) Treatment with the Cav-1 inhibitor M*β*CD (1 mM) recovered K_Ca_2.3 (e) or K_Ca_3.1 (f) levels in ECs treated with PE serum or LPC. Blots shown are representative of three to six experiments performed with three to six different cultures. Bar graphs represent the mean ± SEM. ^∗^*P* < 0.05, ^∗∗^*P* < 0.01.

We compared the effects of soluble serum factors from NNP and NP women on PM-localized K_Ca_2.3 or K_Ca_3.1 levels by incubating HUtMECs in serum from NNP and NP women for 24 hours. K_Ca_2.3 or K_Ca_3.1 proteins were biotinylated at the cell surface and labeled with horseradish peroxidase-conjugated streptavidin. The levels of PM-localized K_Ca_2.3 or K_Ca_3.1 were significantly higher in ECs treated with NP serum than in ECs treated with NNP serum (Figure 1(a)). We then compared the effects of serum from NP and PE women on PM levels of K_Ca_2.3 or K_Ca_3.1 in HUtMECs. The levels of PM-localized K_Ca_2.3 or K_Ca_3.1 proteins were significantly lower in ECs treated with PE serum than in ECs treated with NP serum (Figure 2(b)). In addition, we compared the effect of serum from NNP, NP, and PE women on PM levels of K_Ca_2.3 (Figure 1(c)) or K_Ca_3.1 (Figure 1(d)) in HUtMECs. The levels of PM-localized K_Ca_2.3 or K_Ca_3.1 were not changed in ECs treated with NNP serum, compared to those treated with normal culture medium (CM), and were elevated in ECs treated with NP serum, compared to those treated with NNP serum or CM. Increases in the K_Ca_2.3 or K_Ca_3.1 levels were significantly reduced in ECs treated with PE serum than in ECs treated with NP serum. Since oxidized LDL is among the causative factors to induce endothelial dysfunction in PE, we examined the effects of oxidized LDL on PM levels of K_Ca_2.3 and K_Ca_3.1 by incubating HUtMECs in oxidized LDL containing a culture medium for 24 hours. We found that the levels of PM-localized K_Ca_2.3 or K_Ca_3.1 were significantly reduced upon incubation (Figure 1(e)). Reduced K_Ca_2.3 or K_Ca_3.1 levels by oxidized LDL treatment were reversed by blocking the oxidized LDL receptors using an anti-LOX1 Ab (Figure 1(e)). These results suggest that the expression and localization of K_Ca_2.3 or K_Ca_3.1 in PM are elevated in NP compared to that in NNP, and the elevation was attenuated in PE.

### 3.2. Caveolae and Clathrin Are Involved in the Internalization of K_Ca_2.3 and K_Ca_3.1

The levels of PM-localized proteins, such as ion channels, can be modulated by caveola-dependent internalization. Caveolins, the essential structural elements of caveolae, are suggested to be scaffolding proteins that facilitate the compartmentalization of various signaling molecules or proteins within caveolae. We thus investigated whether caveola-dependent internalization of K_Ca_2.3 or K_Ca_3.1 occurs during pregnancy by examining Cav-1 levels. Cav-1 levels were markedly lower in the ECs treated with NP serum than in ECs treated with NNP serum, and VEGF receptor (VEGFR) inhibition using an anti-VEGFR1 Ab or anti-VEGFR2 Ab enhanced Cav-1 levels in ECs treated with NP serum (Figure 2(a)), indicating that NP serum decreases Cav-1 levels via VEGFR activation. In contrast, Cav-1 levels were markedly higher in ECs treated with PE serum than in ECs treated with NP serum (Figure 2(b)). In addition, LPC, the major component of oxidized LDL, enhanced Cav-1 levels in a concentration-dependent manner (Figure 2(b)). To confirm the presence of Cav-1 in the inner leaflet of the PM, the cell surface was biotinylated with a membrane impermeable agent (NHS-SS-biotin). Biotinylated PM proteins from whole cell lysates were isolated on a streptavidin column and, following elution and SDS-PAGE, were blotted for Cav-1 with anti-Cav-1 Ab. PM localization of Cav-1 was significantly elevated upon treatment with oxidized LDL (Figure 2(c)). We then examined whether caveolae are involved in the regulation of the levels of K_Ca_2.3 or K_Ca_3.1. Colocalization of K_Ca_3.1 with Cav-1 was examined using coimmunoprecipitation. Colocalization of K_Ca_3.1 with Cav-1 was markedly higher in ECs treated with PE serum than in ECs treated with NP serum (Figure 2(d)). Cav-1 inhibition using the Cav-1 inhibitor M*β*CD elevated K_Ca_2.3 (Figure 2(e)) or K_Ca_3.1 (Figure 2(f)) levels in ECs treated with PE serum or LPC. These results suggested that caveola-dependent internalization is involved in regulating the PM localization of K_Ca_2.3 or K_Ca_3.1 during pregnancy. Caveola-dependent internalization process might be delayed in NP, whereas it might be facilitated in PE.

**Figure 3:**
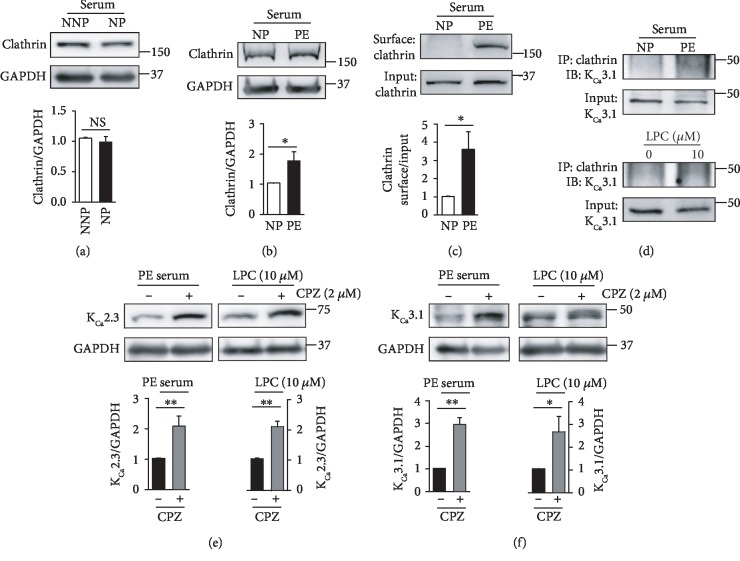
Role of clathrin in the K^+^ channel degradation. (a) Clathrin levels in ECs treated with NNP or NP serum. Alteration in clathrin levels was not found between ECs treated with NNP or NP serum. (b) Clathrin levels in ECs treated with NP or PE serum. (c) Membrane clathrin levels in ECs treated with NP or PE serum. (d) Colocalization of clathrin and K_Ca_3.1 was enhanced by PE serum or LPC. (e, f) Treatment with the clathrin inhibitor CPZ (2 *μ*M) recovered K_Ca_2.3 (e) or K_Ca_3.1 (f) levels in the ECs treated with PE serum or LPC. Blots shown are representative of three to six experiments performed with three to six different cultures. Bar graphs represent the mean ± SEM. ^∗^*P* < 0.05, ^∗∗^*P* < 0.01.

Previously, we reported that K_Ca_3.1 proteins on the PM are internalized via a clathrin-dependent process in Fabry disease [12]. We, therefore, examined whether clathrin-dependent internalization is involved in regulating PM localization of K_Ca_2.3 or K_Ca_3.1 during pregnancy. Clathrin levels were not altered in ECs treated with NNP or NP serum (Figure 3(a)). In contrast, clathrin levels were markedly higher in the ECs treated with PE serum than in ECs treated with the NP serum (Figure 3(b)). Then, clathrin protein was biotinylated at the cell surface. PM clathrin levels were significantly higher in ECs treated with PE serum than in ECs treated with NP serum (Figure 3(c)). Colocalization of K_Ca_3.1 with clathrin was markedly enhanced in ECs treated with PE serum than in ECs treated with NP serum, and LPC markedly increased colocalization of K_Ca_3.1 with clathrin (Figure 3(d)). Furthermore, the clathrin inhibitor chlorpromazine enhanced K_Ca_2.3 (Figure 3(e)) and K_Ca_3.1 levels (Figure 3(f)) in ECs treated with PE serum or LPC. These results suggest that K_Ca_2.3 and K_Ca_3.1 are internalized via a clathrin-dependent process. Clathrin-dependent internalization process might not be affected in NP, whereas it might be facilitated in PE.

### 3.3. A Rab5c- and EEA1-Dependent Process Mediates K_Ca_2.3 or K_Ca_3.1 Degradation

**Figure 4:**
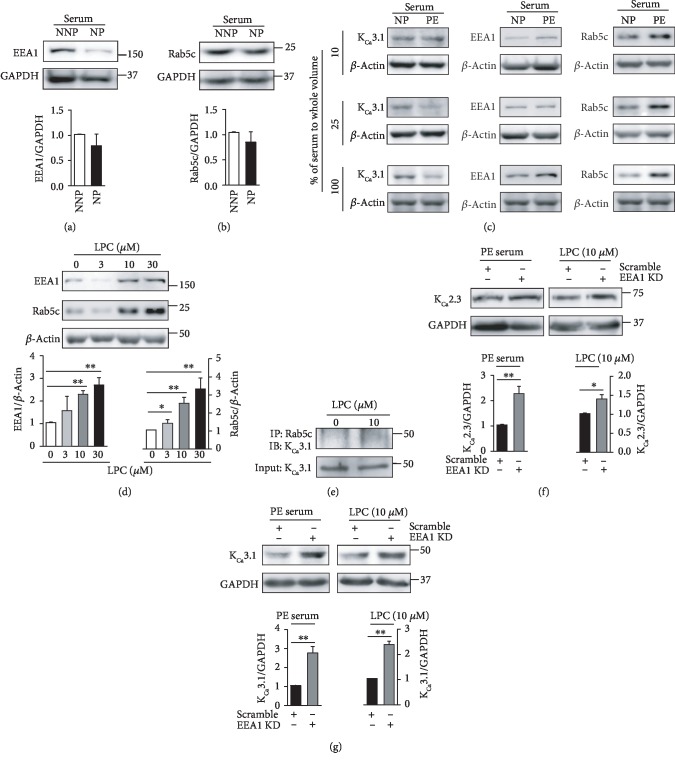
Rab5c- and EEA1-dependent transportation of K_Ca_2.3 and K_Ca_3.1 proteins. (a, b) Levels of EEA1 (a) or Rab5c (b) in ECs treated with NNP or NP serum. (c) PE serum reduced K_Ca_3.1 levels and enhanced Rab5c and EEA1 levels. (d) LPC increased EEA1 and Rab5c levels in a concentration-dependent manner. (e) Coimmunoprecipitation showing the interaction between Rab5c and K_Ca_3.1 in ECs treated with LPC. Input is lysate without primary Ab. (f, g) Treatment with siRNA against EEA1 recovered K_Ca_2.3 (f) or K_Ca_3.1 (g) levels in ECs treated with PE serum or LPC. Blots shown are representative of three to four experiments performed with three to four different cultures. Bar graphs represent the mean ± SEM. ^∗^*P* < 0.05, ^∗∗^*P* < 0.01.

The small GTPases Rab5 and EEA1 play a rate-limiting role in membrane docking or fusion in the early endocytic pathway [17, 18], and our previous study suggested that K_Ca_3.1 is transported into early endosomes via a Rab5c- and EEA1-dependent process in Fabry disease [12]. We thus examined whether a Rab5c- and EEA1-dependent process mediates K_Ca_2.3 and K_Ca_3.1 transportation during pregnancy. Compared to ECs treated with NNP serum, levels of EEA1 (Figure 4(a)) or Rab5c (Figure 4(b)) were slightly decreased in the ECs treated with NP serum. However, significant difference was not found in ECs treated with NNP or NP serum. Then, NP or PE serum was diluted with the culture medium. Compared to the diluted NP serum, the diluted PE serum (10%, 25%, and 100% serum) reduced K_Ca_3.1 levels and increased Rab5c and EEA1 levels in a concentration-dependent manner (Figure 4(c)), indicating that K_Ca_3.1 levels are inversely related to Rab5c and EEA1 levels. In addition, LPC increased Rab5c and EEA1 levels in a concentration-dependent manner (Figure 4(d)). We examined whether Rab5c is involved in the regulation of the levels of K_Ca_3.1 using coimmunoprecipitation. LPC enhanced colocalization of Rab5c with K_Ca_3.1 (Figure 4(e)). We then examined the effect of EEA1 inhibition on LPC-induced downregulation of K_Ca_2.3 or K_Ca_3.1 by using siRNA against EEA1. K_Ca_2.3 (Figure 4(f)) and K_Ca_3.1 (Figure 4(g)) levels, which were reduced by PE serum or LPC, recovered upon EEA1 inhibition. These results suggest that Rab5c and EEA1 are involved in K_Ca_2.3 or K_Ca_3.1 downregulation in PE.

### 3.4. A Redox State Regulates Membrane Levels of K_Ca_2.3 and K_Ca_3.1 during Pregnancy

**Figure 5:**
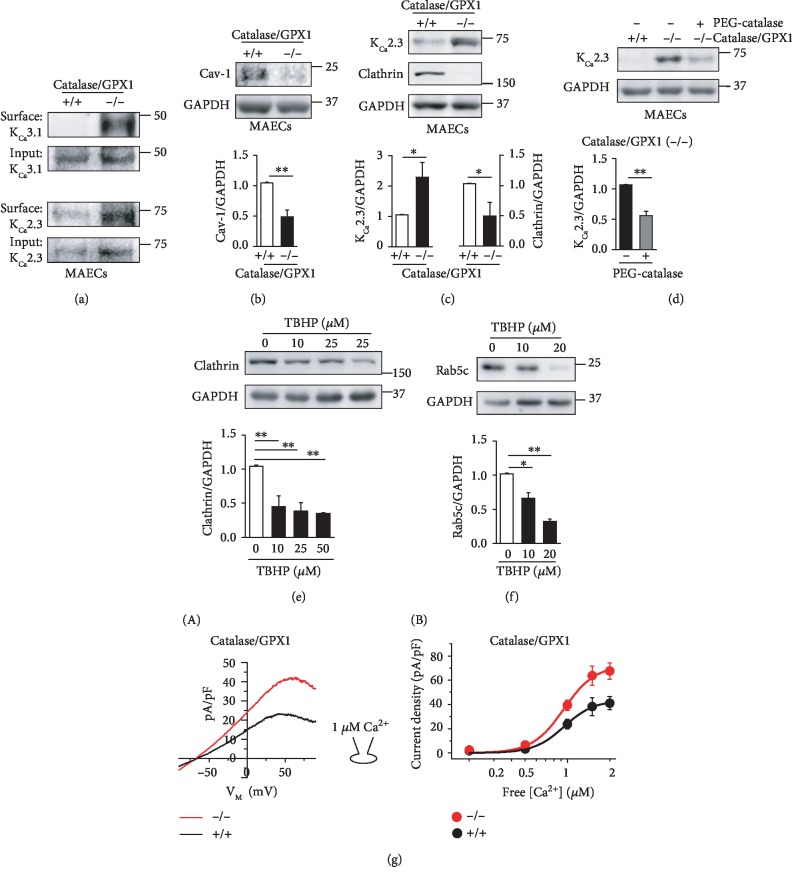
Catalase and GPX1 knockdown slows down K_Ca_2.3 or K_Ca_3.1 endocytosis. (a) PM levels of K_Ca_2.3 or K_Ca_3.1 in MAECs from wild-type and catalase^−/−^/GPX1^−/−^ mice. (b) Cav-1 levels in MAECs from wild-type and catalase^−/−^/GPX1^−/−^ mice. (c) K_Ca_2.3 and clathrin levels in MAECs from wild-type and catalase^−/−^/GPX1^−/−^ mice. (d) Elevated K_Ca_2.3 levels by catalase/GPX1 knockdown were reversed by a membrane permeable catalase, PEG-catalase. (e, f) A H_2_O_2_ donor TBHP reduced clathrin (e) or Rab5c (f) levels in a concentration-dependent manner. (g) K_Ca_3.1 currents activated by loading cells with 1 *μ*M Ca^2+^ (A) and the current-[Ca^2+^]_i_ relationship at +50 mV (B). The current densities at +50 mV are plotted against [Ca^2+^]_i_. Blots shown are representative of three to four experiments performed with three to four different cultures. Bar graphs represent the mean ± SEM. ^∗^*P* < 0.05, ^∗∗^*P* < 0.01.

Previously, we showed that catalase and GPX downregulation increased H_2_O_2_ levels, thereby upregulating K_Ca_2.3 and K_Ca_3.1 in NP [10]. In addition, K_Ca_3.1 levels were elevated in mouse aortic endothelial cells (MAECs) from catalase/GPX1 double knockout (catalase^−/−^/GPX1^−/−^) mice [11]. Thus, we examined whether catalase and GPX1 knockdown affects PM levels of K_Ca_2.3 or K_Ca_3.1 using wild-type and catalase^−/−^/GPX1^−/−^ mice. PM-localized K_Ca_2.3 or K_Ca_3.1 levels were markedly increased in catalase^−/−^/GPX1^−/−^ MAECs, compared to wild-type MAECs (Figure 5(a)). We then compared Cav-1 or clathrin levels in wild-type and catalase^−/−^/GPX1^−/−^ MAECs. In catalase^−/−^/GPX1^−/−^ MAECs, levels of Cav-1 (Figure 5(b)) or clathrin (Figure 5(c)) were markedly reduced, and K_Ca_2.3 levels were elevated (Figure 5(c)). Thus, inverse relation between K_Ca_2.3 and clathrin levels was found. The increase in K_Ca_2.3 levels seen in these cells was reversed by treatment with polyethylene glycol- (PEG-) catalase (Figure 5(d)). Furthermore, a H_2_O_2_ donor TBHP reduced clathrin (Figure 5(e)) and Rab5c (Figure 5(f)) levels in a concentration-dependent manner. These results suggest that catalase and GPX1 downregulation slows down the internalization and trafficking of K_Ca_2.3 and K_Ca_3.1 from PM via H_2_O_2_-mediated pathways.

We then compared K_Ca_3.1 currents in MAECs isolated from wild-type and catalase^−/−^/GPX1^−/−^ mice.  Figure 5(g) shows K_Ca_3.1 currents activated by loading cells with 1 *μ*M Ca^2+^ in the patch pipette (A) and the current-intracellular Ca^2+^ concentration ([Ca^2+^]_i_) relationship at +50 mV (B). The current densities were found to be dependent on [Ca^2+^]_i_. At 0.2, 0.5, 1, 1.5, and 2 *μ*M [Ca^2+^]_i_, the current densities were 1.76 ± 0.21, 3.20 ± 1.06, 23.81 ± 3.96, 38.15 ± 7.46, and 41.10 ± 5.38 pA/pF in wild-type MAECs (*n* = 8), respectively, and 2.39 ± 1.37, 6.42 ± 1.93, 39.33 ± 4.40, 63.72 ± 7.89, and 67.52 ± 6.73 pA/pF in catalase^−/−^/GPX1^−/−^ MAECs (*n* = 8), respectively, suggesting that K_Ca_3.1 currents were significantly enhanced in catalase^−/−^/GPX1^−/−^ MAECs, compared to wild-type MAECs. However, no significant difference was found in EC_50_ between wild-type and catalase^−/−^/GPX1^−/−^ MAECs (948 ± 37 nM [Ca^2+^]_i_ and 934 ± 42 nM [Ca^2+^]_i_, respectively). This implies K_Ca_3.1 upregulation enhances K_Ca_3.1 currents in catalase^−/−^/GPX1^−/−^ MAECs.

**Figure 6:**
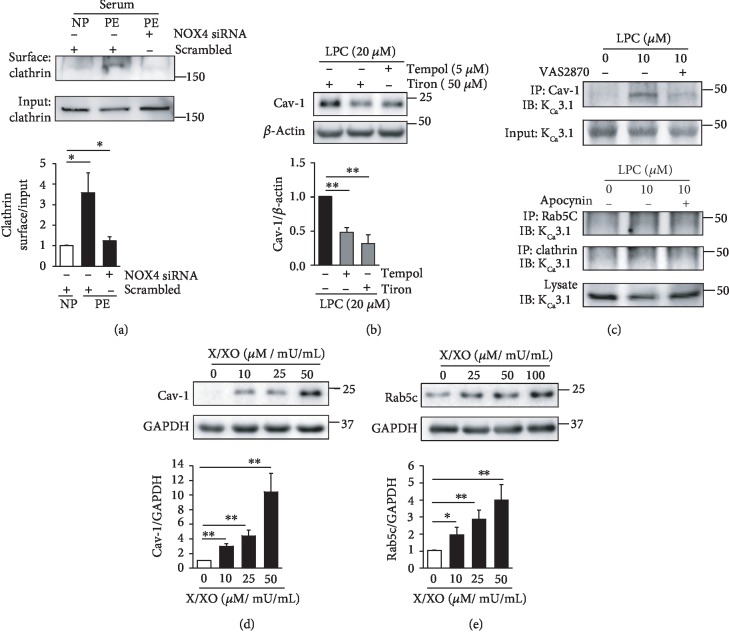
Superoxide facilitates K_Ca_2.3 or K_Ca_3.1 endocytosis. (a) Elevated PM clathrin levels by PE serum were reduced by NOX4 inhibition using NOX4 siRNA. (b) Elevated Cav-1 levels by LPC were reduced by the antioxidants, tempol or tiron. (c) Elevated colocalization of K_Ca_3.1 with Cav-1, clathrin, or Rab5c by LPC was reversed by the treatment with a pan-NOX inhibitor, VAS2870, or apocynin. (d, e) A superoxide donor, X/XO, elevated Cav-1 (d) or Rab5c (e) levels in a concentration-dependent manner. Blots shown are representative of three to four experiments performed with three to four different cultures. Bar graphs represent the mean ± SEM. ^∗^*P* < 0.05, ^∗∗^*P* < 0.01.

On the other hand, we had previously reported that NADPH oxidase 2 (NOX2) and NOX4 upregulation and SOD downregulation enhance superoxide levels, thereby downregulating K_Ca_2.3 and K_Ca_3.1 in PE [10, 19]. We thus examined whether superoxide facilitates the internalization and transportation processes of K_Ca_2.3 and K_Ca_3.1 in PE. Compared to cells treated with NP serum, PM-localized clathrin levels were markedly elevated in ECs treated with PE serum, and the elevation was reversed by the treatment with NOX4 siRNA (Figure 6(a)). LPC elevated Cav-1 levels, and the elevation was reversed by the treatment with the antioxidants, tempol or tiron (Figure 6(b)). LPC increased colocalization of K_Ca_3.1 with Cav-1, clathrin, or Rab5c, which was inhibited by a pan-NOX inhibitor VAS2870 or apocynin (Figure 6(c)). Furthermore, a superoxide donor X/XO increased Cav-1 (Figure 6(d)) or Rab5c (Figure 6(e)) levels in ECs in a concentration-dependent manner. These results suggest that the internalization and transportation processes of K_Ca_2.3 and K_Ca_3.1 facilitated by superoxide contribute to the downregulation of K_Ca_2.3 and K_Ca_3.1 in PE.

## 4. Discussion

**Figure 7:**
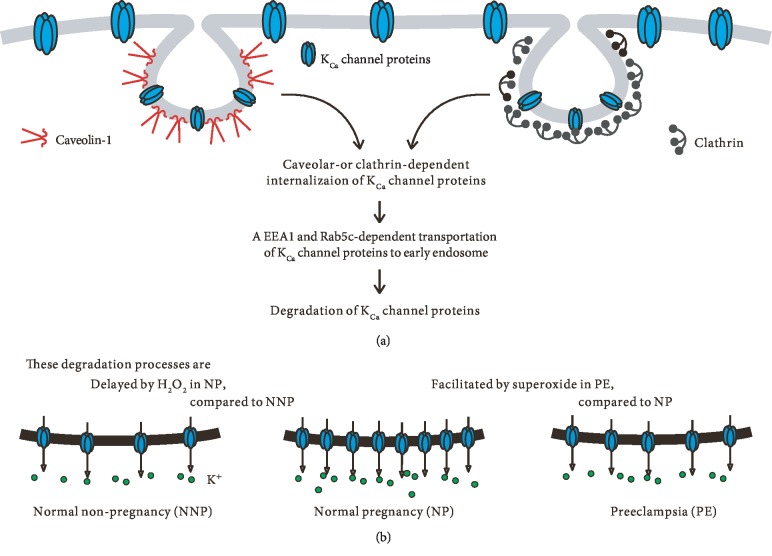
A schematic model for altered redox state-induced modulation of PM-localized K_Ca_2.3 and K_Ca_3.1 in NP and PE. (a) PM-localized K_Ca_2.3 and K_Ca_3.1 are internalized via caveola- or clathrin-dependent processes and transported via a Rab5c- and EEA1-dependent process to early endosome. (b) Compared to NNP, the internalization and transport processes are delayed by H_2_O_2_ in NP, thus elevating PM-localized K_Ca_2.3 and K_Ca_3.1 levels. However, compared to NP, the internalization and transport processes are facilitated in PE, thus reducing PM-localized K_Ca_2.3 and K_Ca_3.1 levels.

In this study, we observed for the first time that PM-localized K_Ca_2.3 and K_Ca_3.1 are internalized via caveola- or clathrin-dependent processes and transported via a Rab5c- and EEA1-dependent process in ECs (Figure 7). Compared to NNP, the internalization and transport processes are delayed in NP, thus elevating PM-localized K_Ca_2.3 and K_Ca_3.1 levels. However, compared to NP, the internalization and transport processes are facilitated in PE, thus reducing PM-localized K_Ca_2.3 and K_Ca_3.1 levels. Soluble factors in PE serum, such as oxidized LDL, might induce internalization of K^+^ channel proteins from the PM via clathrin- or caveola-dependent processes and thereby attenuate the pregnancy-associated K_Ca_2.3 and K_Ca_3.1 upregulation. Since endothelial K_Ca_2.3 and K_Ca_3.1 play important roles in the control of vascular contractility, altered levels of these K^+^ channels might explain the hemodynamic changes seen during the progress of pregnancy, both NP and PE.

K_Ca_2.3 was internalized from PM via a caveola- or clathrin-dependent process. The involvement of caveolae in the internalization of K_Ca_2.3 is consistent with the finding that K_Ca_2.3 is observed in caveolae, Cav-1-rich membrane fractions [20], and that the endocytosis of K_Ca_2.3 from the cell membrane is dependent upon both Cav-1 and dynamin II [21]. In addition, we showed that clathrin is involved in K_Ca_2.3 internalization. Internalized K_Ca_2.3 might be transported via a EEA1- and Rab5-dependent process, since LPC-induced reduced K_Ca_2.3 levels were reversed by siRNA against EEA1 (Figure 4(f)). Similarly, Gao et al. demonstrated the involvement of Rab5-containing endosome in the endocytosis of K_Ca_2.3 from PM [21]. These mechanisms involved in K_Ca_2.3 internalization and transportation were similar to those for K_Ca_3.1 internalization and transportation.

Previously, we demonstrated that PM K_Ca_3.1 proteins are internalized via a clathrin-dependent process and transported in a Rab5c- and EEA1-dependent process in Fabry disease [12]. In addition, we demonstrated that caveolae are involved in K_Ca_3.1 internalization from cell membrane, although endothelial K_Ca_3.1 is suggested to be present in noncaveolar membrane fractions [20, 22]. However, K_Ca_3.1, which is not associated with Cav-1 under baseline conditions, was found to colocalize with Cav-1 during shear stress conditions [23]. In addition, PE serum induced colocalization of K_Ca_3.1 with Cav-1 (Figure 2(d)). Thus, stimulation by serum components or shear stress might lead to colocalization of K_Ca_3.1 and Cav-1. In addition, clathrin-dependent internalization followed by Rab5c- and EEA1-dependent transportation was found to regulate membrane K_Ca_3.1 levels during pregnancy.

Altered redox state might be involved in the regulation of internalization and transportation of K_Ca_2.3 and K_Ca_3.1 in NP and PE. We previously showed that H_2_O_2_ levels are elevated via downregulation of catalase and GPX1 during NP [10]. Catalase and GPX1 knockdown elevated K_Ca_2.3 and K_Ca_3.1 levels in PM (Figure 5(a)) and reduced Cav-1 and clathrin levels (Figures 5(b) and 5(c)), suggesting that H_2_O_2_ elevates PM levels of K_Ca_2.3 and K_Ca_3.1 by slowing down the internalization process of the K^+^ channel proteins from PM. In addition, reduction in Rab5c levels by the H_2_O_2_ donor TBHP suggests that H_2_O_2_ slows down the transportation process of the internalized K_Ca_2.3 and K_Ca_3.1. Thus, reduced degradation of K_Ca_2.3 and K_Ca_3.1 might contribute to the upregulation of these K^+^ channel proteins during NP. In contrast, superoxide might facilitate the internalization and transportation processes of K_Ca_2.3 and K_Ca_3.1 from cell membrane. NOX2 and NOX4 upregulation and SOD downregulation enhanced superoxide levels, thereby downregulating K_Ca_2.3 and K_Ca_3.1 in PE [10].

Reduction in Cav-1 levels by NP serum was reversed by anti-VEGFR Abs, suggesting that soluble serum factors, such as VEGF, might inhibit caveola-dependent internalization of K_Ca_2.3 and K_Ca_3.1 through VEGFR activation during NP. Since serum levels of the soluble VEGF inhibitor, sFlt-1 are increased in PE, VEGFR activation might be suppressed during PE. Inhibition of VEGFR activation might contribute to the increase in Cav-1 levels by PE serum. In addition, cell membrane localization of Cav-1 and clathrin was enhanced by the treatment with oxidized LDL or LPC, and LPC increased colocalization of clathrin with K_Ca_3.1. Thus, VEGFR inhibition or oxidized LDL might facilitate internalization of K_Ca_2.3 and K_Ca_3.1, thereby reducing membrane levels of these K^+^ channels during PE.

Oxidative stress is increased during pregnancy (in both NP and PE) [24–26]. ROS reduces NO bioavailability by interacting directly with NO [27–29], suggesting that NO-induced vasorelaxation might be impaired during pregnancy. Since K_Ca_2.3 and K_Ca_3.1 activation induces NO-independent vasorelaxation by evoking hyperpolarization of VSMCs, K_Ca_2.3 and K_Ca_3.1 upregulation may compensate for decreased NO signaling, as shown in vascular aging [11]. K_Ca_2.3 and K_Ca_3.1 upregulation in NP suggests that a similar compensation occurs in NP [10]. In contrast, ROS and superoxide production has been suggested to be more in PE than in NP, and superoxide decreased levels of endothelial NO synthases [30]. These results suggest that the impairment of NO-induced vasorelaxation might be greater in PE than in NP. In addition to the impaired NO-induced vasorelaxation, reduced K_Ca_2.3 and K_Ca_3.1 upregulation suggests that NO-independent, endothelium-dependent relaxation is also impaired in PE [10].

The present findings suggest that PM-localized K_Ca_2.3 and K_Ca_3.1 levels are regulated by similar internalization and transportation processes, and the regulation of the processes is affected by an altered redox state during NP and PE. We identified Cav-1, clathrin, Rab5c, and EEA1 as critical components in the regulation of PM-localized K_Ca_2.3 and K_Ca_3.1 levels during pregnancy. Inhibiting Cav-1, clathrin, or EEA1 using siRNAs against these components led to the recovery of PM-localized K_Ca_2.3 and K_Ca_3.1 levels, suggesting that siRNAs against these components can be used to treat endothelial dysfunction. To the best of our knowledge, this is the first study to reveal the mechanisms underlying the regulation of membrane K_Ca_2.3 and K_Ca_3.1 levels during pregnancy.

## Figures and Tables

**Table 1:** Blood pressure levels of subject groups.

Group	Normal nonpregnancy (*n* = 8)	Normal pregnancy (*n* = 15)	Preeclamptic pregnancy (*n* = 12)
Systolic blood pressure (mmHg)	115.0 ± 1.0	117.7 ± 1.7	153.0 ± 3.2
Diastolic blood pressure (mmHg)	74.0 ± 1.7	77.4 ± 2.1	97.7 ± 2.9

Values shown are mean ± SEM and exclusively composed of plasma donors.

## Data Availability

The data used to support the findings of this study are available from the corresponding author upon request.

## Abbreviations

Ab: Antibody
Cav-1: Caveolin-1
CM: Culture medium
CPZ: Chlorpromazine
EC: Endothelial cell
EEA1: Early endosome antigen-1
HUtMEC: Human uterine microvascular EC
LDL: Low-density lipoprotein
LOX: Lectin-like oxidized LDL receptor
LPC: Lysophosphatidylcholine
MAECs: Mouse aortic endothelial cells
M*β*CD: Methyl-*β*-cyclodextrin
NNP: Normal nonpregnancy
NO: Nitric oxide
NOX: NADPH oxidase
NP: Normal pregnancy
PE: Preeclampsia
PM: Plasma membrane
sFlt-1: Soluble fms-like tyrosine kinase-1
VEGF: Vascular endothelial growth factor
VEGFR: VEGF receptor.
